# Evidence-based Decision Making: Infectious Disease Modeling Training for Policymakers in East Africa

**DOI:** 10.5334/aogh.4383

**Published:** 2024-03-22

**Authors:** Sylvia K. Ofori, Emmanuelle A. Dankwa, Emmanuel Ngwakongnwi, Alemayehu Amberbir, Abebe Bekele, Megan B. Murray, Yonatan H. Grad, Caroline O. Buckee, Bethany L. Hedt-Gauthier

**Affiliations:** 1 Center for Communicable Disease Dynamics, Harvard T.H. Chan School of Public Health, Boston, MA, USA; 2 Institute of Global Health Equity Research, University of Global Health Equity, Kigali, Rwanda; 3 School of Medicine, University of Global Health Equity, Kigali, Rwanda; 4 Department of Global Health and Social Medicine, Harvard Medical School, Boston, MA, USA; 5 Department of Immunology and Infectious Diseases, Harvard T.H. Chan School of Public Health, Boston, MA, USA

**Keywords:** Africa, Capacity building, Epidemiological models, Infectious diseases, Policy

## Abstract

**Background:**

Mathematical modeling of infectious diseases is an important decision-making tool for outbreak control. However, in Africa, limited expertise reduces the use and impact of these tools on policy. Therefore, there is a need to build capacity in Africa for the use of mathematical modeling to inform policy. Here we describe our experience implementing a mathematical modeling training program for public health professionals in East Africa.

**Methods:**

We used a deliverable-driven and learning-by-doing model to introduce trainees to the mathematical modeling of infectious diseases. The training comprised two two-week in-person sessions and a practicum where trainees received intensive mentorship. Trainees evaluated the content and structure of the course at the end of each week, and this feedback informed the strategy for subsequent weeks.

**Findings:**

Out of 875 applications from 38 countries, we selected ten trainees from three countries – Rwanda (6), Kenya (2), and Uganda (2) – with guidance from an advisory committee. Nine trainees were based at government institutions and one at an academic organization. Participants gained skills in developing models to answer questions of interest and critically appraising modeling studies. At the end of the training, trainees prepared policy briefs summarizing their modeling study findings. These were presented at a dissemination event to policymakers, researchers, and program managers. All trainees indicated they would recommend the course to colleagues and rated the quality of the training with a median score of 9/10.

**Conclusions:**

Mathematical modeling training programs for public health professionals in Africa can be an effective tool for research capacity building and policy support to mitigate infectious disease burden and forecast resources. Overall, the course was successful, owing to a combination of factors, including institutional support, trainees’ commitment, intensive mentorship, a diverse trainee pool, and regular evaluations.

## Background

Infectious diseases are significant contributors to the global burden of diseases, especially in Africa, and there are often substantial social and economic costs associated with the public health measures implemented to control them [1,2]. For example, public health measures during the COVID-19 pandemic, such as lockdowns, disrupted access to essential health services and supply systems and led to the shutting down of businesses in Africa [3,4]. Mathematical models are a group of tools used by researchers to forecast the spread of infectious diseases, predict the outbreak size and outcomes, and assess the impact of nonpharmaceutical interventions – i.e. social distancing, use of facial masks, and testing – and pharmaceutical interventions – i.e. vaccinations and pharmacotherapy [5,6,7]. Thus, these models play an important role in providing evidence to support decision-making related to preventing or treating infectious diseases [8].

The use of mathematical models in public health policy is well-adapted to the decision-making process for epidemic and endemic diseases in other regions [8]. For example, global health organizations such as the World Health Organization (WHO) and Joint United Nations Programme on HIV/AIDS (UNAIDS), among others, have relied on findings from mathematical modeling studies to make policies around intervention selection and vaccine optimization strategies for diseases like Influenza, Ebola, HIV, and COVID-19 [8]. These models are often not fully integrated into the decision-making process in low- and middle-income countries, particularly in Africa, in part due to the limited mathematical modeling capacity, difficulty in building model complexity, lack of trust in the findings given the many assumptions, and the unwillingness of authorities to apply findings [9,10].

Several African countries demonstrate global leadership in infectious disease mathematical modeling. For example, South Africa has mathematical modeling units such as South Africa’s Modelling and Simulation Hub Africa (MASHA), the South African Centre of Excellence in Epidemiological Modelling and Analysis (SACEMA), and the Centre for Infectious Disease and Epidemiology Research (CIDER). These institutions have a long-standing reputation for supporting the government with evidence-based policy. However, there is a geographical disparity in the mathematical modeling outputs in Africa, with South Africa and Morocco having the most capacity and Central and East African countries like Chad, Rwanda, and Uganda having limited outputs [11]. A 2022 report identified training and mentorship as key approaches to strengthening mathematical modeling capacity in Africa [12]. This report recommended that trainings should be structured as short courses, formal training with continuous support as students develop models, and project-driven training. Other researchers have also highlighted the need for infectious disease modeling training in the continent [10,11].

In 2023, our team implemented the Mathematical Modeling for Infectious Diseases Planning Course targeting public health professionals in East Africa. Here, we describe the design and implementation, including challenges, successes, and lessons learned to inform other infectious disease training programs in the region.

## Overview of the Training Program

This training was developed through a partnership between the University of Global Health Equity (UGHE), Rwanda, and the Center for Communicable Disease Dynamics (CCDD), Harvard Chan School of Public Health, USA. The course reflected the core principles of the Intermediate Operational Research Training (IORT) led by Partners In Health/Rwanda and Harvard Medical School [13] and the WHO’s Structured Operational Research Training Initiative (SORT-IT) courses [14,15,16]. These principles include using a hands-on, deliverable-driven approach with a) didactic training with in-person sessions; b) all trainees working on a project related to their work that reinforces the didactic milestones; c) progression through the project, requiring trainees to achieve milestones to remain in the course; and d) intensive mentorship built into the program to ensure trainees’ and their projects’ success. We contrast our mathematical modeling training to the IORT in Table 1 and provide specific details of the mathematical modeling course below.

**Table 1:** Comparing the IORT Course and the UGHE/Harvard Mathematical Modeling (MM) training.


AREA	IORT	MM TRAINING

Approach	Deliverable-driven approach to training with intensive mentorship during practicum and in-person sessions.	Deliverable-driven approach to training with intensive mentorship during practicum and in-person sessions.

Target trainees	Program and clinical staff	Infectious disease public health professionals

Frequency/length of training	Varied, first cohort was 2-day modules, every 4–6 weeks to 3 6-day modules, every 2–5 months, with 3 milestones.	2 2-week training of four weeks, occurring three months apart.

Deliverable	A manuscript submitted to a peer-reviewed journal.	A policy brief and an abstract for conferences.

Training advertisement	Advertised within the priority organizations and professional networks.	Advertised within professional networks of the training team and the Training Advisory Committee (TAC), and on social media on LinkedIn, Facebook, and Twitter.

Trainee selection and number	Applicants applied in pairs and were selected based on the applications’ strengths and the strategic value of the research.	Applicants applied as individuals or in pairs and were selected upon consultation with TAC based on country, gender, strength of application, and organizational priorities.

Training format	Lectures, break-out writing sessions with mentorship, plenary sessions for group feedback and a practicum period to implement skills.	Lectures, class activities to work on their models, presentations for peer and expert feedback, and practicum to implement skills.

Facilitation and Mentorship	2 mentors, 4 project mentors, and 5 junior mentors for the first cohort. In-person mentorship was offered during training and practicums.	3 core trainers for all four weeks of the training. Three technical experts- one for the 2 weeks of the first session, 1 for each week of the second session of the training. Two training advisors. In-person intensive mentorship offered during training and remote mentorship during practicum.

Projects	Simple, descriptive projects, which could be completed within 8 months using routine program data. Trainees were mentored from peer review process to journal acceptance.	Infectious disease program priority questions using parameter values identified from literature, which could be completed during the duration of the training.

Data analysis	Data was analyzed in STATA.	Mathematical model was developed and analyzed in Berkeley Madonna, a beginner user friendly differential equation solving tool [17].

Costs	Full scholarship provided to participants including tuition fees, travel, expenses and full accommodation, publication, and conference attendance support, and research fieldwork related costs.	Full scholarship provided to participants including tuition fees, travel, expenses and full accommodation, conference attendance support, and stipend at the end of each session.

Monitoring and evaluation	Participants’ appraisal of the training workshop about their background, motivation, and structure of the course.	Participants evaluated the structure and content of the training at the end of each week of training to inform subsequent sessions. Also, trainees provided overall evaluation of the course at the end of the training including training logistics.


## Training Structure

### Training advisory committee

We convened a training advisory committee (TAC) made up of five individuals with experience in supporting efforts in strengthening mathematical modeling and analytical capacities in Africa. The TAC was set up to provide oversight to the training team. Their role included advising on the curriculum and course structure, recruitment strategies, and facilitating dissemination of deliverables from the training.

### Training goals

Our overall goals were to strengthen the mathematical modeling capacity to enhance their use in decision-making and effective communication of modeling outputs to policymakers. Specifically, we hoped for trainees to:

– Understand the fundamental concepts of infectious disease dynamics and mathematical modeling,
– Know the types of models and their use in public health for different infectious diseases,
– Identify research questions suitable for mathematical modeling,
– Critically review infectious disease models and develop them to assess the effectiveness of pharmaceutical and nonpharmaceutical interventions,
– Build mathematical models for assessing the effectiveness of pharmaceutical and nonpharmaceutical interventions, and
– Prepare policy briefs and abstracts for conference submissions as main deliverables. We did not include manuscripts/publications as a primary output given the limited time for the training and the focus of the training on using mathematical models to support policymaking.

### Course structure and curriculum overview

We provided four weeks of in-person training at the UGHE campus in Northern Rwanda. These sessions were divided into two two-week sessions with three months of practicum in between sessions. The first in-person session occurred March 13–24, 2023, and the second in-person session occurred July 24–August 2, 2023. This course was longer than the three weeks of in-person training for the IORT because the content was more challenging.

The course was divided into four weeks: a) Week One: Introduction to Mathematical Modeling b) Week Two: Building a Mathematical Model, c) Week Three: Incorporating Complexity into Mathematical Models, and d) Week Four: Disseminating and Communicating Model Findings (Table 2).

**Table 2:** Overview of curriculum.


WEEK	TITLE	LEARNING OUTCOMES	MILESTONES

One	Introduction to infectious disease modeling	Know basic definitions of infectious disease modeling and the types of models and approaches. Simulate a basic mathematical model and extend it to answer basic questions and assess interventions. Know the sources of literature for summarizing evidence of group projects.	Suggest a topic for the training project and propose research questions. Present a literature review of the chosen topic. Download and install Berkeley Madonna.

Two	Building mathematical models to answer research questions	Develop mathematical models for projects. Write corresponding mathematical model equations. Identify preliminary parameter values for models.	Draft of the mathematical model. Test-run model in Berkeley Madonna to generate curves. Presentation of the background of the project, the natural history of select disease, and the compartmental model for the project. Finalize questions and scenarios to be modeled.

Three	Incorporating complexity into mathematical models	Peer critique of preliminary results of other teams. Incorporate scenarios and/or interventions into models. Evaluate and summarize intervention effects. Review of three articles to understand how researchers assess interventions in mathematical models, and how results are presented. Perform sanity checks of modeling results.	Identify the key messages of model findings. Project presentations to peers and invited experts for feedback.

Four	Communicating and disseminating model findings	Develop the skills for effectively communicating model results to different audiences. Explain differences between policy briefs and research articles. Draft policy briefs. Ability to find relevant conferences and develop abstracts.	Prepare policy briefs. Prepare abstracts for academic conferences. Presentation of model findings to invited guests from different infectious disease backgrounds.


The in-person sessions included lectures focusing on concepts of infectious disease and modeling methods. We included several activities each day for trainees to apply the concepts to their projects to reinforce the knowledge and skills gained. Group and panel discussions and plenary sessions offered trainees the opportunity to learn from and engage with other experts in the field. We encouraged peer learning by including several slots for teams to present their project’s progress to their peers and receive feedback. During practicum periods, participants received hands-on and intensive mentorship from the training team.

#### Trainee projects and practicums

To foster peer learning and improve government/academic partnerships, trainees worked in pairs with each team coming from a single country and focused on a specific disease and question. A pre-training orientation introduced trainees to the course and during the orientation, trainees identified topics of interest and received input from the training team. The final topic was selected before the first in-person session. Trainees completed the practicum at their home site and met virtually with the training team biweekly to report progress and receive mentorship to progress on their projects. Training team members were available in person a week before and a week after each session to provide in-person support to Rwanda-based trainees.

#### Development of course materials

All training materials, except the modeling software, were developed by the course faculty. Most of the materials were originally created for the training, with some being adapted from faculty’s course materials prepared for other contexts. During the in-person sessions, the course faculty met every 1–2 days to modify materials based on trainees’ needs, progress, and feedback. We used Berkeley Madonna as the modeling software because of its ease of use, and straightforward syntax; Berkeley Madonna provided free software licenses for trainees and trainers [17]. The training materials have been made freely available online to serve as reference material for future training: https://ccdd.hsph.harvard.edu/2023-mathematical-modeling-course/.

#### Trainers’ profile

The core training team was made up of three individuals who provided intense hands-on support during the in-person and practicum sessions. The three core trainers were PhD-level statisticians and epidemiologists who have extensive experience in infectious disease research and curriculum development at a global level. In addition, there were three senior-level technical experts—one for the first session and two for the last session—with expertise in mathematical modeling of various infectious diseases including tuberculosis, malaria, Ebola, and COVID-19. They provided expert knowledge and support on the natural history of disease areas of training projects, interventions to be assessed, and model structures. The core team and trainers were supported by other faculty from partnering institutions. Two members of the core training team mentored the trainees during practicum as they finalized their objectives and model structures and incorporated complexity into the models, including assessing interventions and sensitivity analysis.

## Participant Selection

### Application process

The call for applications was advertised within professional networks and social media handles like LinkedIn, Twitter, and Facebook. A complete application included questions about background in infectious diseases, research areas of interest, the relevance of training to their career, a letter of support from a supervisor, and curriculum vitae (see appendix). Applicants were encouraged to apply in pairs, with one applicant from a quantitative background and the other with a program/policy background. Following the advice from the TAC, individual applications were welcomed, and those who applied as individuals were eventually paired based on common research interests and backgrounds. The application docket required applicants to select two topics from a table of suggested infectious disease and intervention areas based on recommendations from the TAC and the research expertise of the core training team.

### Participant selection process

Eligible trainees were public health professionals working in infectious disease programs or research in Africa, with an interest in using mathematical models and a good command of the English language. We targeted this group to provide professional development, foster inter-country learning, and improve quantitative skills in public health practice. As this was the first cohort of the training, and with few facilitators, the training slots were limited to ten individuals divided into five teams.

The selection committee, including the core training team and the TAC, reviewed applications in three stages. We first assessed the completeness based on application instructions and the timing of submission. The second stage included assessing eligibility based on the quality of the application and professional background to identify 20 finalists. The TAC was consulted to select ten successful applicants based on the strength of the application and organizational priorities and made final decisions also ensuring a range of representation from different countries and by gender.

### Background of applicants and selected trainees

**Figure 1:**
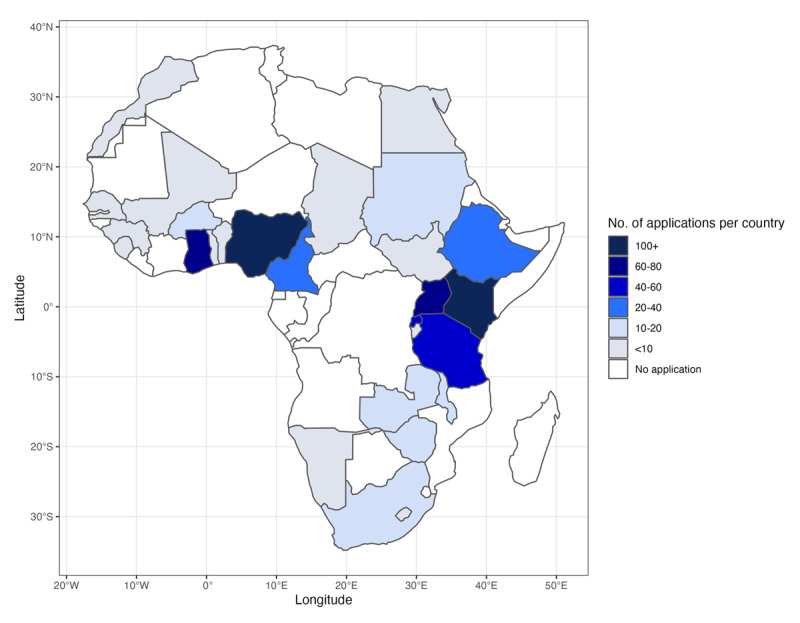
Number of applications per African country.

We received 875 applications, 650 (74%) of which were complete and screened. Most of the complete applications were from Kenya (n = 116, 18%), Nigeria (n = 103, 16%), and Uganda (n = 75, 12%) (Figure 1). Ten participants— four females and six males—were selected. Six trainees were from Rwanda, two from Uganda, and two from Kenya (Table 3). Most trainees (7/10) had master’s degrees, and 9/10 were from government institutions. Although our goal was to have each team have a person with a quantitative background and the other with a policy/program background, one team had both members with the same background. However, this did not alter the dynamics of the team, as one member had more quantitative training.

**Table 3:** Description of trainees.


VARIABLE	TRAINEES (n = 10 (%))

Gender	

Male	6 (60%)

Female	4 (40%)

Highest education	

Bachelors	1 (10%)

Masters	7 (70%)

Doctorate	2 (20%)

Country	

Rwanda	6 (60%)

Uganda	2 (20%)

Kenya	2 (20%)

Type of organization	

Government	9 (90%)

Academia	1 (10%)

Background	

Research	6 (60%)

Policy/program	4 (40%)

Ever taken a course in infectious disease epidemiology	

Yes	5 (50%)

No	5 (50%)

Ever taken a course in mathematical modeling of infectious diseases?	

No	10 (100%)

Relevance of course to current work	

Extremely relevant	7 (70%)

Very relevant	3 (30%)


### Projects of trainees

The five projects the trainees worked on were: i) the impact of vaccination and surveillance on Ebola in Rwanda, ii) the impact of preventive therapy on tuberculosis incidence in Nakuru county, Kenya, iii) the role of enhanced surveillance and vaccination on Ebola outcomes in Mubende District, Uganda, iv) impact of Paxlovid, an antiviral drug, on long COVID and COVID-19 mortality in Rwanda, and v) impact of direct-acting antivirals on Hepatitis C elimination targets in Rwanda.

## Training Evaluation and Dissemination

### Training evaluation

The application packet included questions about research background, how skills obtained from the training would impact their career, and commitment to training. Trainees’ expectations and experience with the training and feedback on the content were assessed using a survey administered at the course’s beginning and at the end of each week to allow trainers to modify subsequent sessions. We also solicited feedback on the structure and logistics at the end of the course using a survey and trainer-trainee meetings with each team. The surveys were semi-structured and included multiple-choice, linear scales, and open-ended questions (see appendix). We calculated the frequency of responses and median scores of linear scales, generated bar charts, and open-ended questions were analyzed thematically.

**Figure 2:**
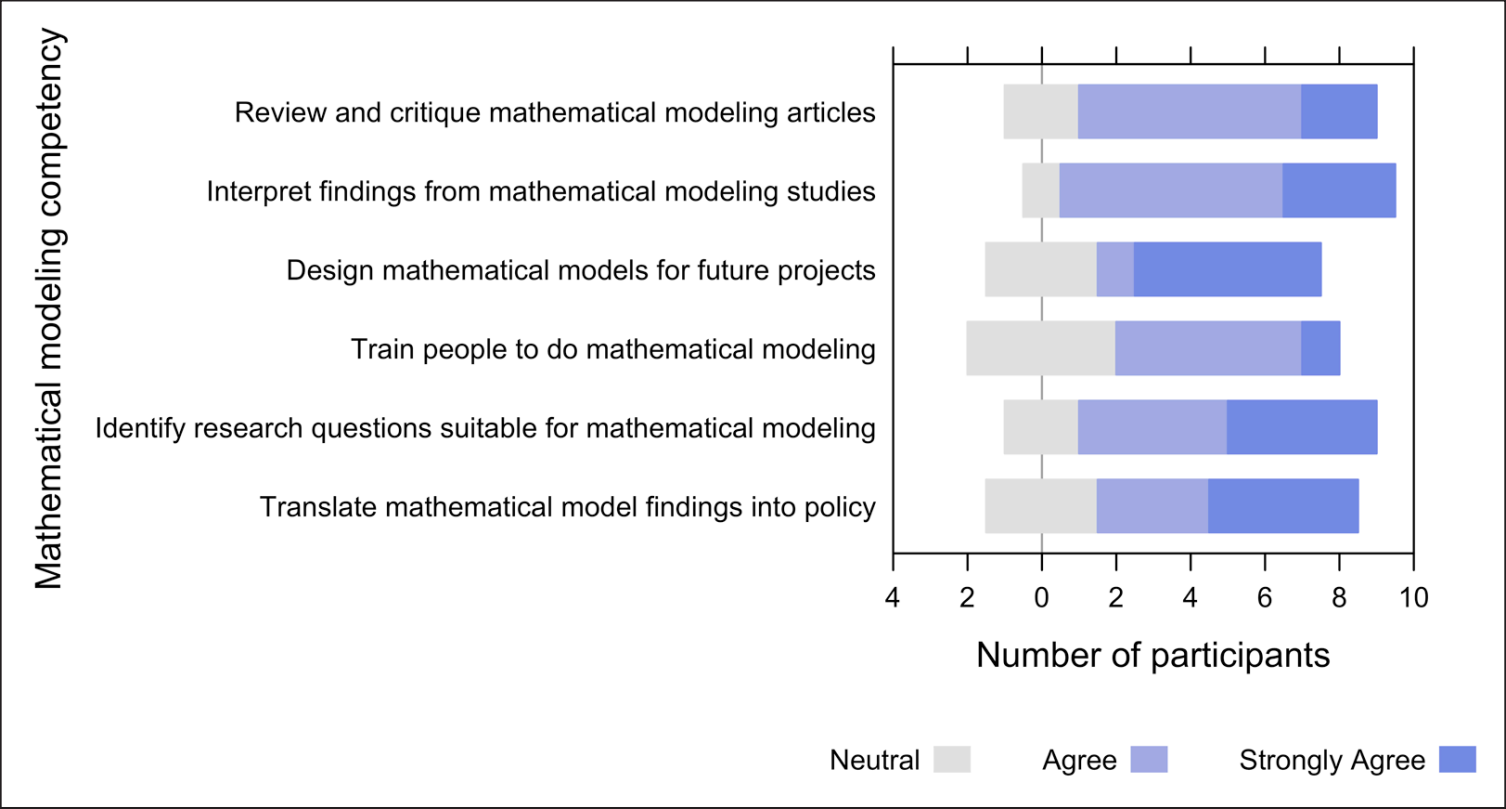
Assessment of mathematical modeling capacity competency using data from the ten participants.

Five trainees (50%) had previously taken a course in infectious disease epidemiology, but none had ever taken a course in mathematical modeling. When asked about the relevance of the training to their field of work, 70% deemed the training to be “extremely relevant” (Table 3). We assessed the extent to which mathematical modeling competency was attained using the indicators in Figure 2. Six trainees agreed they could confidently review and critique mathematical modeling studies and five strongly agreed they could confidently design their compartmental models for future projects at the end of the training. Moreover, four strongly agreed that they could translate mathematical modeling findings into policy. For the overall quality of the training, on a scale of 1–10 (1 = very poor, 10 = excellent), the median score was 9 (see Appendix). All trainees completed the course. All trainees indicated they would recommend the course to their colleagues.

**Figure 3:**
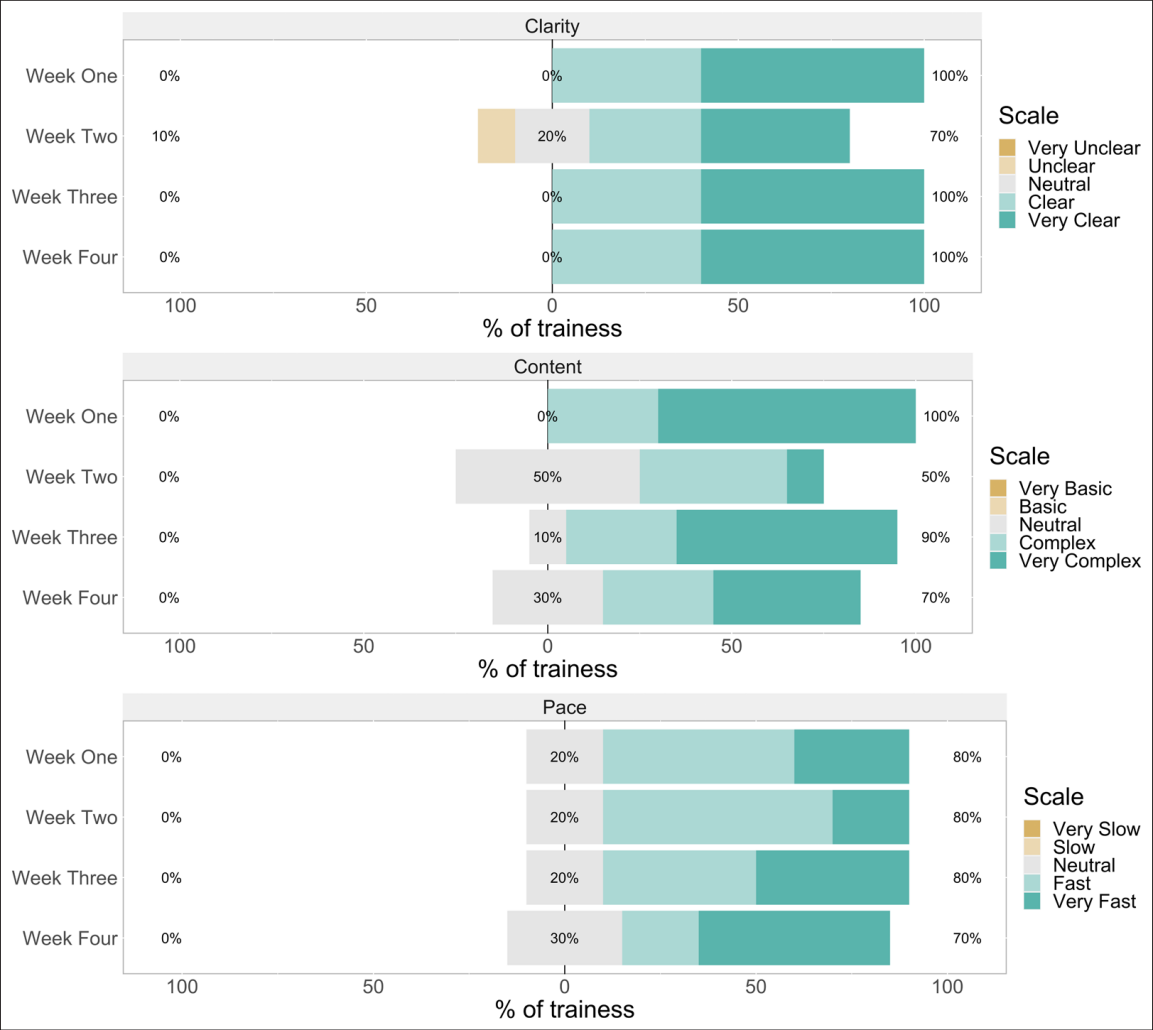
Bar charts of rating of clarity (upper panel), content (middle panel), and pace (lower panel) of ***lectures*** at the end of each training week (n = 10).

**Figure 4:**
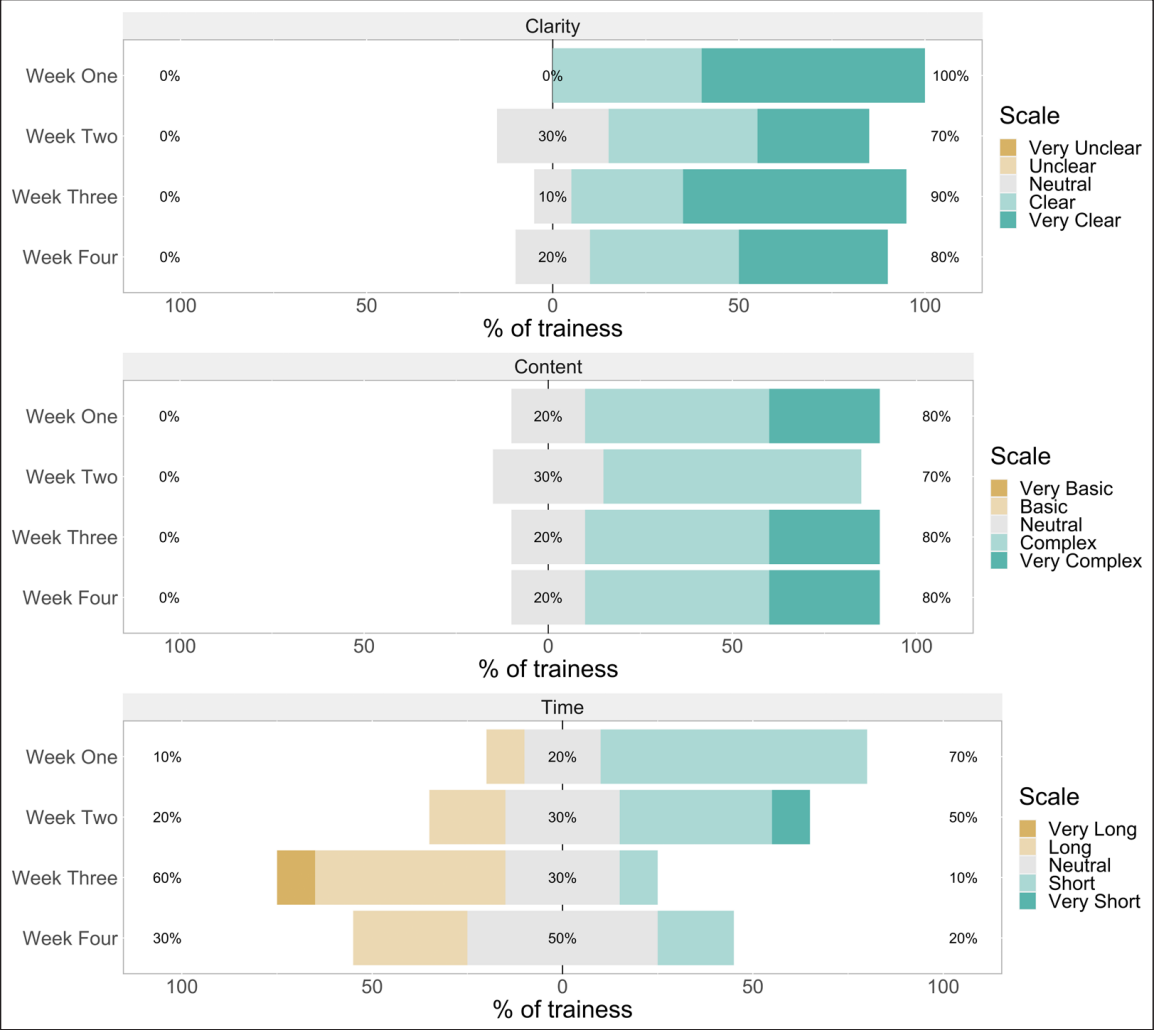
Bar charts of rating of clarity (upper panel), content (middle panel), and time allocation (lower panel) of ***activities*** at the end of each training week (n = 10).

Trainees were asked to rate the clarity, pace, and content of lectures at the end of each training week (Figure 3). Six participants indicated the lectures were very clear in weeks one, three, and four. The highest number of participants (n = 5) indicated that the pace of the lectures was very fast in week four. None of the trainees indicated that the pace was slow or very slow in the four weeks of training. Most trainees rated the content of lectures as very complex in week 1 (n = 7), followed by week 3 (n = 6). The quality of class activities was also rated on a similar scale (Figure 4). Six trainees rated the clarity of activities as “very clear” in weeks one and three. The rating of the time allocated to class activities being short was observed in weeks one (n = 7) and two (n = 4). Three trainees rated the content of activities as very complex in weeks one, three, and four, while the majority (7/10) assigned a rating of complex in week two.

There are several limitations to our training evaluation. First, we have not yet conducted a longitudinal follow-up to assess the application of the skills gained in their professional roles and if policy briefs impacted decision-making, but we anticipate doing so in the future. Second, while these surveys were anonymous, given the small number of participants, the responses may be subject to desirability bias. For that reason, we have paid attention both to the scaled responses and the open-ended replies. Third, in the goal of keeping the surveys brief, not all possible areas were assessed. For example, we did not ask about the trainees’ confidence that the skills gained would be useful in their work settings.

### Dissemination

The training deliverables were policy briefs and abstracts distributed among relevant stakeholders. Trainees presented their projects to guests, including policymakers, program managers, and researchers, at the graduation ceremony, where trainees were presented with certificates. Four teams submitted their abstracts to the 2023 edition of the Conference on Public Health in Africa, and two projects are being supported for publication.

### Course cost

All training-related cost was covered for all sessions. The scholarship included transportation to the training location, meals, accommodation, and flights for non-Rwanda trainees. Trainees also received a stipend of 100 USD per person at the end of each session to help cover costs incurred for the training, including public transport to airports for non-Rwanda trainees and transport to pick-up locations. Trainee teams whose abstracts are accepted for a conference presentation would receive financial support to attend and present their projects. Berkeley Madonna team also supported the training in kind by providing all trainees, two of the UGHE-affiliated training advisors with perpetual licenses, and four of the trainers with one-year licenses. The trainees also received approval from their supervisors to offer their time in-kind to participate in the training.

## Successes, Challenges, and Lessons Learned From the Training

### Strengths and facilitators of success

A variety of factors contributed to the success and strengths of the training, where success is defined as achieving the deliverables by the course end date and 100% course completion rate. A core feature of this program, and critical for trainees’ success, is the intensive mentorship provided, the hands-on approach, and the regular communication between trainees and trainers, especially during the practicum. There were many practical sessions, which provided trainees the space to implement the concepts learned and to receive feedback. We created a WhatsApp group to facilitate easier communication between trainers and trainees, as well as knowledge sharing and networking among trainees. Additionally, during in-person sessions, trainers were available for consultations at the end of each training day. Trainees used these consultations to seek clarity on concepts taught or on project-specific challenges encountered. Selecting trainees from three countries offered cross-country networking opportunities and greatly enriched the diversity of perspectives in class discussions, helping trainees to learn from others’ systems. Moreover, the variety in the infectious disease expertise of trainees (tuberculosis, COVID-19, Ebola, and Hepatitis C) further contributed to the diversity of perspectives.

In addition, instrumental support from trainees’ institutions was important. The application packet required applicants to confirm that their supervisors supported their applications and to submit a letter of support from the employers. Applicants’ eligibility therefore depended on supervisors and employers providing approval for trainees to attend the training. Requiring trainees to develop their models for their institutions’ priority questions served as a motivation for employers to approve trainees’ time away to attend training. We also received institutional support from the Rwanda Biomedical Center, which was the home institution for five trainees.

Another facilitator of success was the trainees’ commitment, demonstrated by completing milestones typically on time and earning certificates of completion amidst competing commitments from their full-time work. To ensure fewer distractions for trainees, especially those based in Rwanda, UGHE’s Butaro campus was chosen as the training location, as the campus is located in a rural community 80 miles north of Rwanda’s capital Kigali. Trainees rated their satisfaction with the location of the training with a median score of 6.5, on a scale of 1–10 (1 = not satisfied, 10 = very satisfied). The score may be due to transportation to the location, which is remote and accessed through unpaved roads, as transportation also received a median score of 6.5 (See Appendix).

The pre-training orientation, held two months before the first training session, was also integral for success as it helped prepare trainees for the intensive training period by providing foundational information on the course structure, expected outcomes and software tools to be employed. The orientation was also an opportunity to propose topics of interest and receive feedback on topic feasibility from trainers. Finally, the weekly evaluation of the clarity, pace, and content of the training, helped improve the training program as it was implemented, continually structuring delivery to better suit trainees’ needs.

### Challenges

Here we highlight some challenges in the course’s planning and implementation to inform other mathematical modeling training programs. First, the demand for the course, demonstrated through the volume of applications (875 applications), far exceeded the number anticipated by the training team and the number of slots available. Consequently, processing these applications took longer than planned and required substantial effort from two trainers. Additional support for program coordination might have alleviated this issue. Second, given that most of the content was developed originally for this training, the time and effort required to prepare the course content was substantial. We were able to draw from previous teaching experience and courses of our training team, but we still spent considerable time developing content appropriate for these trainees, that had practical sessions integrated, and that applied concepts to the trainees’ project. One benefit is that we can leverage this first offering’s materials for future courses. However, even these materials need to be tailored to specific trainee populations and projects and doing so is non-trivial.

Furthermore, even with planning and dedicated trainer resources, providing intensive mentorship to meet trainees’ interests and demands was challenging. Mentorship required mentors to be flexible with their schedules to ensure they were readily available to respond to participants’ questions and troubleshoot errors in their codes. The training could also have benefited from full-time in-country mentorship support, especially during the practicum. We attempted to address the issue of in-country support by having training mentors based in Rwanda a week before and after each session to provide in-person mentorship. However, the non-Rwanda trainees did not benefit from these in-Rwanda resources.

Third, not all five teams could be supported to translate their findings to publication, given the limited bandwidth of the trainers and the limited availability of some trainees to invest the time required to realize a full research article. For these reasons, only two projects are currently being developed for publication. Lastly, using the 2-2 training model meant many new concepts were introduced to trainees over a short period and trainees had limited time to apply these concepts to their projects during the training, making the training very intensive. Trainees expressed a preference for a 2-2-2 training model, adding a third two-week session to be able to spread out the concepts over a longer period (see Appendix). This would offer many advantages but with the obvious consequence of needing additional resources to implement and the bigger commitment for trainees to be absent from their work and family obligations.

### Recommendations

Based on our experience with planning and implementing the UGHE/Harvard Mathematical Modeling for Infectious Diseases Planning Course, we recommend the following as avenues to improve and scale infectious disease modeling training in Africa.

### Funding

The high demand for infectious disease modeling training should be matched with more funding opportunities to support this kind of training. Notably, there has recently been an increasing number of calls from international non-governmental organizations to fund these kinds of training endeavors, although these funding calls are spontaneous and irregular. Making training more sustainable demands that funding streams are continuous and established, such as that which may come from the national/local government budget or a national/local health agency [18,19]. When trainings are locally sponsored, it is more likely that training goals will serve to answer infectious disease questions of local priority and thus would be more relevant. We acknowledge that in low- or middle-income countries, national/local budgets may be limited [20,21]. We, therefore, encourage funding partnerships between national/local governments and non-governmental organizations to achieve the goal of a continuous funding stream while ensuring that funded training tackles questions of mutual interest [22]. We also advise funders to consider funding programs that are smaller, longer, and with more intensive mentorship over models, seemingly prioritized by funders, that train more people on superficial levels.

### In-country expertise

Improving the sustainability of infectious disease modeling training also requires more country-based faculty training experts [13]. This will reduce the cost of sponsoring international trainers and ease collaborations between national health policymakers and modeling researchers, given that locally-based researchers have a better context and understanding of local infectious disease issues. In-country faculty, ideally in a local university or research institute, will also provide more opportunities for longer-term training, such as at the master’s or PhD level. Moreover, some of the trainees indicated their interest in further training in infectious disease modeling. We acknowledge however that Africa-based faculty-level expertise in infectious disease modeling is currently limited; however, if trainings such as the one we have organized are more frequent, the situation can be expected to improve in a few years [11].

### Geography

We recommend that as much as possible, future trainings of this kind target individuals from across Africa [23]. We limited our participant pool to East Africa, mainly because of resource limitations; however, the interest (Figure 1) and need [11] for infectious disease modeling are not limited to a particular geographical region of the continent.

### Orientation

We recommend that future trainings include an orientation session to offer trainees conversance with course structure and expectations before the training begins [24]. There should be enough time between the orientation and the start of the training to allow trainees to adjust to training expectations and to allow trainers to adapt content to trainees’ needs as may be identified during the orientation.

### Training time

We recommend that future trainings consider the need to balance the time allocated to teaching concepts and having hands-on sessions where trainees apply the concepts learned. Future training should consider an extended model, for example, having three in-person sessions with longer practicums in between.

## Conclusions

We have described the 2023 UGHE/Harvard Mathematical Modeling for Infectious Diseases Planning Course which trained ten East African public health practitioners to develop infectious disease models to answer questions of interest in their local contexts. Overall, the course was successful, owing to a combination of factors, including institutional support, trainees’ commitment, intensive mentorship, a diverse trainee pool, and regular evaluations. The main challenges faced were the limited capacity relative to the need and the limited time available for training. Given our experiences with planning and implementing the course, we recommend: i) more funding opportunities for similar training in Africa, ii) training is carried out by, or involves in-country experts; iii) future training target participants from across Africa, include a pre-training orientation and consider a balance between the time for teaching concepts and having hands-on sessions.

## Additional file

The additional file for this article can be found as follows:

10.5334/aogh.4383.s1 Appendix. Appendix A1–A5.
