# Supplementary material for: Evidence-based Decision Making: Infectious Disease Modeling Training for Policymakers in East Africa
Source: Ann Glob Health. 2024 Mar 22;90(1):22. doi: 10.5334/aogh.4383 (PMC10959131; doi:10.5334/aogh.4383)

Evidence-based decision making: Infectious disease modeling training for policymakers in East Africa

Appendix

Sylvia K. Ofori[1*], Emmanuelle A. Dankwa[1], Emmanuel Ngwakongnwi[2], Alemayehu Amberbir[2], Abebe Bekele[3], Megan B. Murray[4], Yonatan H. Grad[5], Caroline O. Buckee[1], Bethany L. Hedt-Gauthier[4]

1 Center for Communicable Disease Dynamics, Harvard T.H. Chan School of Public Health, Boston, MA, USA
2 Institute of Global Health Equity Research, University of Global Health Equity, Kigali, Rwanda
3 School of Medicine, University of Global Health Equity, Kigali, Rwanda
4 Department of Global Health and Social Medicine, Harvard Medical School, Boston, MA, USA
5 Department of Immunology and Infectious Diseases, Harvard T.H. Chan School of Public Health, Boston, MA, USA

* Correspondence to Sylvia Ofori; sylviaofori@hsph.harvard.edu

A1: UGHE/Harvard Mathematical Modeling Training Application document

2022-2023 University of Global Health Equity/Harvard Mathematical Modeling for Infectious Diseases

The University of Global Health Equity (UGHE), in collaboration with Harvard University, is hosting a mathematical modeling course for public health practitioners and researchers in Africa. The training will be held at the UGHE campus in Rwanda for four weeks of training over the course of one year.

The training will introduce participants to mathematical models for infectious diseases and their applications in answering questions relevant to public health programs and policy. Participants will also gain the skills of critically appraising modeling studies to identify findings that are relevant for practice. The program is designed for participants to have hands-on experience with mathematical modeling; trainees design their models from concepts taught in class and use models to answer research questions for their group projects.

The training requires a year-long commitment with sessions alternating between in-person, which occurs approximately every four months, and remote practicum sessions. During the in-person sessions, we will use a combination of lectures, group discussions, computer practical sessions, and guest lectures to teach trainees the concepts of mathematical modeling. At the end of each in-person session, each team will be expected to achieve smaller milestones while receiving intensive mentorship from the training team both in-person and remotely. A certificate of participation will be awarded to trainees upon successful completion of the training.

The dates for the in-person sessions are as follows:

- First session (Week 1): December 5-9, 2022.
- Second session (Week 2&3): March 20-31, 2023 (anticipated dates, to be confirmed)
- Third session (Week 4): July 24-28, 2023 (anticipated dates, to be confirmed)

We invite applications from individuals with backgrounds in epidemiology, biostatistics, policy, and program management who work in infectious disease research and/or program implementation. Applicants are encouraged, but not required, to apply in teams of two individuals with one person from a policy/program background and a second from a research/analyst background. To be eligible for consideration, applicants must have experience using Microsoft Excel and have a good command of the English language. No computer programming experience is required.

In-Rwanda transportation, accommodation, and related expenses will be provided during the training. Travel scholarships up to $1000/person per training session will be available for individuals selected outside of Rwanda to cover the costs of travel to and from Rwanda.

To apply, please complete the attached application form and submit the forms along with your CV and letter of support from your supervisor. Please note that the deadline for the training team to receive all application materials is November 5, 2022.

Application packets must be submitted as one PDF document to Sylvia Ofori at sylviaofori@hsph.harvard.edu.

2022-2023 Harvard/University of Global Health Equity
Mathematical Modeling for Infectious Disease Planning

Training Application

Please apply to the training by completing and submitting this form. Note, applicants are encouraged, but not required, to apply as teams of two individuals with one person from a policy/program background and a second from a research/analyst background:

-If applying as a team, two separate applications must be completed by team members and submitted together in one PDF packet to the email provided.

- If applying as an individual, send the single application to the email provided and the selection committee will attempt to identify a suitable teammate from the applicant pool.

The application is completed once we receive: the electronic application forms, both applicant CVs (where applicable), and letters of support from the applicant's supervisors.

The deadline for submission of all applications to be considered for the training is 11/05/2022.

Name:
Name of your co-applicant (recommended, but not required):

Email:
Phone number:
Job title/position:
Organization:

Gender: ( ) M ( ) F ( ) Non-binary ( ) Prefer not to specify
Highest education level: ( ) Bachelors ( ) Masters ( ) Doctorate ( ) Other:
Primary city and country of residence:

Do you have experience in infectious disease program implementation or research?
( ) Yes ( ) No

Background information (Please note these are not requirements for your applications to be considered. Providing the following details will help us gauge the needs of applicants.)

Do you have any publications?
( ) Yes ( ) No

If yes, please provide the citations to your publications below:

i) ……………………………………………………….

ii) ……………………………………………………
iii) ………………………………………………………

What is your level of proficiency with Excel spreadsheets?
( ) Beginner ( ) Intermediate ( ) Advanced ( ) Expert

Have you taken a calculus course? ( ) Yes ( ) No
Have you taken linear algebra courses? ( ) Yes ( ) No

All participants will be expected to have access to a working laptop. Can you confirm?
( ) I have access to a working laptop
( ) I can get access to a working laptop by November 2022

<u>Requirements for selection:</u> Please attest to the following.

1. **I understand that if I am selected for the training, I must attend three in-person training sessions.**
   ( ) Yes Applicant's signature: …………….

2. **I understand that if I am selected, I will be expected to dedicate a couple of hours a week to my project with a team member during the practicum session.**
   ( ) Yes Applicant's signature: ……………….

3. **I have permission from my supervisor to participate in this training and will submit a letter of support along with the application materials.**
   ( ) Yes Applicant's signature: ………………………...

4. **Indicate your preferred disease area and topic(s) among the following by checking with "X", the cell that best suits your research interest. (<u>Please select a maximum of two cells</u>.)**

**Disease area**	**Vaccinations**	**Nonpharmaceutical interventions**	**Testing and surveillance**	**Other**
**COVID-19**	☐	☐	☐	☐
**Measles**	☐	☐	☐	☐
**Rubella**	☐	☐	☐	☐
**Influenza**	☐	☐	☐	☐
**Malaria**	☐	☐	☐	☐
**Yellow fever**	☐	☐	☐	☐
**Ebola**	☐	☐	☐	☐
**Dengue**	☐	☐	☐	☐
**Tuberculosis**	☐	☐	☐	☐
**STDs**	☐	☐	☐	☐
**Other (please specify):**	☐	☐	☐	☐

5. **Describe your research interest in relation to your chosen topic, elaborating on specific experiences that make the topic the best fit for you. (Maximum of 200 words)**

6. **What role will the skills you obtain from this training play in your work? (Maximum of 100 words)**

7. **Briefly describe your research experience. (Maximum of 100 words)**

8. **Do you perceive any hindrances to your participation and completion of this training?**

A2: Example of the weekly course evaluation survey

1. **How did this week's training increase your knowledge of incorporating complexity into infectious disease modeling?** *
Mark only one answer.

No change 1 2 3 4 5 6 7 8 9 10 Major improvement

2. **How would you rate the clarity of the lectures?** *
Mark only one answer.

Very unclear 1 2 3 4 5 6 7 8 9 10 Very Clear

3. **How would you rate the content of the lectures?** *
Mark only one answer.

Very basic 1 2 3 4 5 6 7 8 9 10 Very Complex

4. **How would you rate the pace of the lectures?** *
Mark only one answer.

Too slow 1 2 3 4 5 6 7 8 9 10 Too Fast

5. **How would you rate the clarity of the hands-on activities?** *
Mark only one answer.

Very unclear 1 2 3 4 5 6 7 8 9 10 Very Clear

6. **How would you rate the content of the hands-on activities?** *
Mark only one answer.

Very basic 1 2 3 4 5 6 7 8 9 10 Very Complex

7. **How would you rate the time allocated to the hands-on activities?** *
Mark only one answer.

Too Short 1 2 3 4 5 6 7 8 9 10 Too Long

8. **Which of the lectures did you find most useful and why?** *

9. **Which of the activities did you find most useful and why?** *

10. **What was your favorite aspect of the course this week?** *

11. **What recommendations do you have to improve the course content and/or structure?** *

12. **Are there any concepts you would like us to revisit?** *

13. **Any other comments?** *

A3: Overall course evaluation

Thank you for completing the weekly evaluations. For this week, you will be providing an additional evaluation of other aspects of the training. Please note that your responses are completely anonymous.

* Indicates required question

---

1. How satisfied are you with the duration of two weeks per session? *
Mark only one answer.

Very unsatisfied 1 2 3 4 5 6 7 8 9 10 Very Satisfied

2. In the future, what would you say is the best structure for this course? *
Mark only one answer.

- 1-2-1 training model (One week of training, followed by a practicum, then two weeks of training, then a practicum, and one more week of training
- 2-2 training model (Two weeks of training, followed by a practicum, and another two weeks of training)
- 2-2-2 training model (Two weeks of training, followed by a practicum, another two weeks of training, and a practicum, and two weeks of training

3. How would you rate the duration of the practicum (3 months between the two sessions)? *
Mark only one answer.

Very Short 1 2 3 4 5 6 7 8 9 10 Very Long

4. How would you rate your experience working with a team member with a complimentary background (i.e., policy/program manager versus quantitative background)? *
Mark only one answer.

Very Bad 1 2 3 4 5 6 7 8 9 10 Excellent

5. How satisfied were you with the length of the training day (8:30 am to 5 pm)? *
Mark only one answer.

Very Unsatisfied 1 2 3 4 5 6 7 8 9 10 Very Satisfied

6. How satisfied were you with the location of the training (i.e., Butaro/UGHE)? *
Mark only one answer.

Not Satisfied 1 2 3 4 5 6 7 8 9 10 Very Satisfied

7. How satisfied were you with transportation to Butaro/Kigali? *
Mark only one answer.

Not Satisfied 1 2 3 4 5 6 7 8 9 10 Very Satisfied

8. How satisfied were you with hospitality during the training? *
Mark only one answer.

Not Satisfied 1 2 3 4 5 6 7 8 9 10 Very Satisfied

9. How satisfied were you with the extracurricular activities (e.g. Zumba and scavenger hunt) incorporated into the training? *
Mark only one answer.

Not Satisfied 1 2 3 4 5 6 7 8 9 10 Very Satisfied

10. How satisfied were you with the use of Berkeley Madonna as the software package for mathematical modeling? *
Mark only one answer.

Not Satisfied 1 2 3 4 5 6 7 8 9 10 Very Satisfied

11. Do you have any additional comments or suggestions to improve the course structure and general activities?

____

12. How do the following apply to you in regard to the objectives of the training?

Mark only one oval per row.

	Strongly disagree	Disagree	Neutral	Agree	Strongly agree
I can confidently review and critique mathematical modeling articles.	⬭	⬭	⬭	⬭	⬭
I can interpret findings from mathematical modeling studies.	⬭	⬭	⬭	⬭	⬭
I can design mathematical models for future projects.	⬭	⬭	⬭	⬭	⬭
I can train people to do mathematical modeling.	⬭	⬭	⬭	⬭	⬭
I can identify research questions suitable for mathematical modeling.	⬭	⬭	⬭	⬭	⬭
I can translate mathematical modeling findings into policy.	⬭	⬭	⬭	⬭	⬭

13. **How would you rate your confidence in sharing your knowledge of infectious disease modeling with peers?** *

Mark only one answer.

Not Satisfied 1 2 3 4 5 6 7 8 9 10 Very Satisfied

14. **What comments or recommendations do you have related to the course content and/or objectives?** *

15. **How did you hear about this training?**

Mark only one oval.

- LinkedIn
- Twitter
- Facebook
- Direct email
- From a colleague or peer

16. **How would you rate the overall quality of the training?** *

Mark only one answer.

Very Poor 1 2 3 4 5 6 7 8 9 10 Excellent

17. **Would you recommend the training to colleagues?** *

Mark only one oval.

Yes

No

18. **Any other comments?** *

A4: Median scores and range for selected evaluation questions

Question	Median	Range
Satisfaction with duration of practicum	5	1-9
Satisfaction with the location of training	6.5	4-10
Satisfaction with transportation	6.5	3-10
Satisfaction with hospitality	8.5	7-10
Overall quality of the training	9	7-10

A5: In the future, what would you say is the best structure for this course?

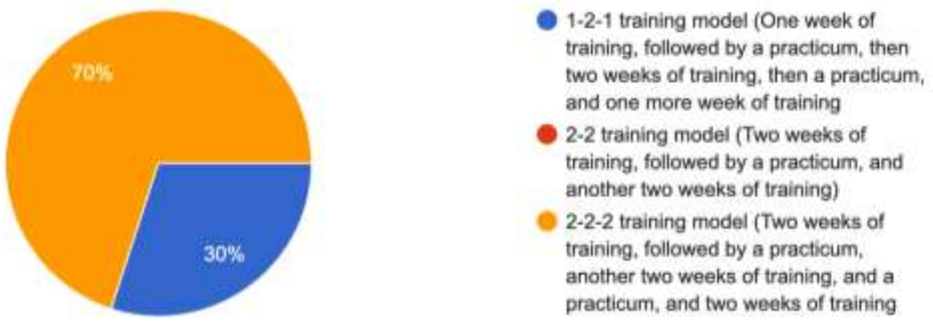

Supplement: Appendix. — Appendix A1–A5. [file agh-90-1-4383-s1.pdf]
